# Differences in brain activations between micro- and macro-expressions based on electroencephalography

**DOI:** 10.3389/fnins.2022.903448

**Published:** 2022-09-12

**Authors:** Xingcong Zhao, Ying Liu, Tong Chen, Shiyuan Wang, Jiejia Chen, Linwei Wang, Guangyuan Liu

**Affiliations:** ^1^School of Electronic and Information Engineering, Southwest University, Chongqing, China; ^2^Key Laboratory of Cognition and Personality, Ministry of Education, Southwest University, Chongqing, China; ^3^School of Music, Southwest University, Chongqing, China

**Keywords:** micro-expressions, macro-expressions, emotion, electroencephalography (EEG), expression inhibition

## Abstract

Micro-expressions can reflect an individual’s subjective emotions and true mental state and are widely used in the fields of mental health, justice, law enforcement, intelligence, and security. However, the current approach based on image and expert assessment-based micro-expression recognition technology has limitations such as limited application scenarios and time consumption. Therefore, to overcome these limitations, this study is the first to explore the brain mechanisms of micro-expressions and their differences from macro-expressions from a neuroscientific perspective. This can be a foundation for micro-expression recognition based on EEG signals. We designed a real-time supervision and emotional expression suppression (SEES) experimental paradigm to synchronously collect facial expressions and electroencephalograms. Electroencephalogram signals were analyzed at the scalp and source levels to determine the temporal and spatial neural patterns of micro- and macro-expressions. We found that micro-expressions were more strongly activated in the premotor cortex, supplementary motor cortex, and middle frontal gyrus in frontal regions under positive emotions than macro-expressions. Under negative emotions, micro-expressions were more weakly activated in the somatosensory cortex and corneal gyrus regions than macro-expressions. The activation of the right temporoparietal junction (rTPJ) was stronger in micro-expressions under positive than negative emotions. The reason for this difference is that the pathways of facial control are different; the production of micro-expressions under positive emotion is dependent on the control of the face, while micro-expressions under negative emotions are more dependent on the intensity of the emotion.

## Introduction

In human interpersonal interactions, the face’s complex musculature and direct relationship with the processing and perception of emotions in the brain make it a dynamic canvas on which humans transmit their emotional states and infer those of others (Porter et al., 2012). However, individuals may suppress or hide their true emotional expressions in certain social situations, which complicates the interpretation of facial expressions. Brief facial expressions revealed under such voluntary manipulation are often referred to as micro-expressions (Ekman and Rosenberg, 2005; Ekman, 2009). As instantaneous expressions within 1/2 to 1/25 of a second, micro-expressions are faint and difficult to recognize by the naked eye, but they are believed to reflect a person’s true intent, especially those of hostile nature (ten Brinke et al., 2012a). Micro-expressions are similar to macro-expressions and have basic, discrete types of emotional expressions, such as disgust, anger, fear, sadness, happiness, or surprise, that reveal the emotions that a person may be attempting to hide (Ekman, 2003; Porter and Brinke, 2008). Therefore, micro-expressions can provide essential behavioral clues for lie detection, which has been increasingly used in fields such as national security, judicial systems, medical clinics, and social interaction research.

However, the neural mechanisms of micro-expression remains unclear, which affects the further development of micro-expression recognition applications. The current recognition of micro-expressions rely heavily on image recognition technology (Peng et al., 2017; Zhang et al., 2018; Wang et al., 2021) and expert assessment (Yan et al., 2013, 2014). Image recognition technology refers to the technology of object recognition of human face images using various machine learning (Wang et al., 2015; Liu et al., 2016) and deep learning algorithms (Verma et al., 2020; Wang et al., 2021) to recognize micro-expressions. Expert assessment refers to an assessment method in which a professionally trained micro-expression recognition expert manually judges micro-expressions. The disadvantages of image recognition technology are limited application scenarios (Goh et al., 2020), such as those with covered facial micro-expressions (e.g., wearing a mask during the pandemic), insufficient illumination (Xu et al., 2017), and special people (e.g., patients with facial paralysis). The main problem with expert assessment is subjectivity, which is not only time-consuming but also has a low correctness rate (Monaro et al., 2022). Therefore, exploring the neural mechanisms of micro-expressions can lay the foundation for micro-expression recognition based on physiological signals. This can help to overcome the limitations of micro-expression recognition and broader application scenarios for micro-expression recognition.

The inhibition hypothesis proposed by Ekman (Malatesta, 1985) suggests that micro-expressions are produced by competition between the cortical and subcortical pathways in emotional arousal, which involves both emotional arousal and voluntary cognitive control processes. When an emotion is triggered, the subcortical brain regions project a strong involuntary signal from the amygdala to the facial nucleus. The individual subsequently recruits the voluntary motor cortex to conceal this response, sending a signal to suppress their expression in a socially and culturally acceptable manner. This means that the cortical pathways, including the temporal cortex, primary motor cortex, ventrolateral premotor cortex, and supplementary motor area, can evaluate and make decisions regarding facial expressions and subsequently recruit motor areas that directly control voluntary facial movements (Paiva-Silva et al., 2016). This view can be considered as the basic assumption of the neural perspective of micro-expressions (Frank and Svetieva, 2015).

Therefore, this is the first study that aims to explore the brain mechanisms underlying micro-expressions and their differences from macro-expressions from a neuroscientific perspective. Micro-expressions have the characteristics of spontaneity, short duration, and low intensity. Therefore, the millisecond temporal resolution of EEG technology can rapidly capture brain activity when micro-expressions occur. Moreover, it has the advantage of being non-invasive and low-risk, and a large number of studies have used EEG techniques to examine the brain mechanisms underlying macro-expression generation (Recio et al., 2014; Shangguan et al., 2019; Yuan et al., 2021). For example, using EEG techniques, Recio et al. found that, compared to happy expressions, angry expressions came along with greater allocation of processing resources for the inhibition of the preactivated motor plan (N2), and the updating of a new one (P3)(Recio et al., 2014). Shangguan et al. also used EEG to examine the brain mechanisms underlying the production of happy and angry facial expressions. They found that happiness and anger did not differ during the motor-preparation phase. The difference in amplitudes between N2 and P3 showed that the inhibition and reprogramming costs of anger were greater than those of happiness (Shangguan et al., 2019). Thus, it is evident that the high temporal resolution of EEG technology is substantially advantageous in studying the brain mechanisms underlying the generation of micro- and macro-expressions.

Currently, the suppression-elicitation and lying-leakage paradigms are the approaches typically used for micro-expression elicitation (ten Brinke et al., 2012b; Yan et al., 2014). The lying-leakage method (Ekman and Friesen, 1974; Frank and Ekman, 1997), although more ecologically valid, is problematic because the micro-expression occurrence rate is quite low and EEG studies require a certain number of occurrences before analysis. In contrast, the suppression-elicitation paradigm requires participants to maintain neutral facial expressions while watching a video and eliciting strong emotions. Their performance is related to an experimental reward that increases their motivation to hide their true emotions in facial expressions (Yan et al., 2013, 2014). Video-induced high emotional intensity increased the occurrence of micro-expressions; 109 MEs were detected among 1000 facial expressions (approximately 11%). However, micro-expressions mostly occur in interpersonal situations; thus, we added a real-time supervision module to the suppression-elicitation paradigm to improve the ecological validity of this method. Consequently, the subjects and supervisors participated simultaneously in the experiments, providing simulated social supervision. We named this improved paradigm real-time supervision and emotional expression suppression (SEES) experimental paradigm.

In summary, we designed a paradigm (SEES) to investigate the neural mechanisms via EEG synchronization with a high-speed camera for the difference between micro and macro-expressions under voluntary conditions at the scalp and source levels. Meanwhile, we examined the effect of different types of emotions (i.e., whether the neural mechanisms of micro-expressions are different under positive and negative emotions).

## Materials and methods

### Participants

There were 80 self-reported, right-handed participants in the current study. The participants were healthy and did not consume psychoactive substances. Those at risk for depression were excluded (Beck Depression Inventory score of > 18). Of these 80 participants, 78 exhibited at least one instance each of happy and fearful expressions during positive and negative video clips. Thus, the final sample comprised 78 participants (age range: 17–22 years, 23 males, 45 females). All participants provided written informed consent and the ethics committee of Southwest University approved the study.

### Materials

We chose videos of amusement, fear, and neutral emotions as emotional stimuli to elicit sufficient, relatively pure facial expressions that were not surrounded by various unemotional facial movements. Chinese comedy film clips and variety shows were used for the amusement videos, since native cultural factors may affect elicitation in emotional experiments (Zheng et al., 2017). Classic scary films were used for fear videos. The criteria for selecting the video materials were as follows: (a) the length of the video was < 3 min to avoid visual fatigue, (b) the materials were easily understood and did not require excessive thinking, and (c) the materials should elicit the expression of a single desired target emotion (e.g., urge to laugh or express fear). Based on these criteria, we manually selected 35 online videos as the emotional materials. We requested 20 participants (not part of the formal experiment) to assess the valence of these videos and rate the intensity on a seven-point Likert scale; six points and above were the criteria for selection. We selected seven videos as the elicitation material for experimentation, including three positive (eliciting laughter urges, scale 6.05 ± 0.83), one neutral, and three negative (eliciting fear expression, scale 6.13 ± 0.92) video clips.

### Experimental design

**FIGURE 1:**
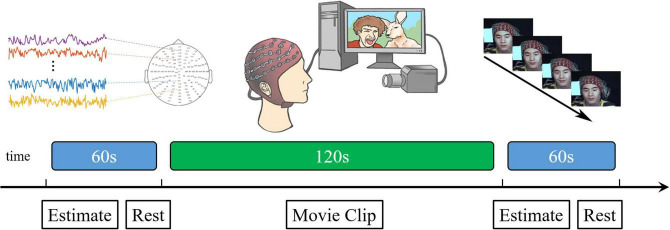
Schematic representation of the presentation of a single block video material.

To put the participants into strong motivation to inhibit facial expressions and improve the ecological validity, we increased the pressure (simulated social supervision situation) into the classic micro-expressions suppression-elicitation paradigm (SEES experimental paradigm) to strongly motivate participants to inhibit facial expressions and improve ecological validity. The participants and supervisors participated simultaneously in the experiment. Participants were seated approximately 1 m from a 23-inch screen, behind which there were two cameras: a high-speed recording camera (90 frames/s) and a real-time surveillance camera on a tripod. The supervisor was seated approximately 1 m to the left of the participant to observe facial expressions through a monitor in real time. The participant was aware of the supervisor’s presence. Participants and supervisors were divided by a curtain to ensure that the movement of supervisors did not affect the participant’s attention. EEG signals were recorded from 128 active electrodes using a Biosemi Active system. A labview-based synchronization system was developed to synchronize the EEG acquisition device accurately with a high-speed camera. We ensured that the EEG signal was accurately synchronized with the acquisition of facial images by using the same trigger simultaneously to generate time stamps on the camera recording and Biosemi Active system (see Figure 1).

Participants were seated in a comfortable chair in a silent room at a temperature of 24–26°C. Participants were instructed to concentrate on the video clips and neutralize their facial expressions. Additionally, they were informed that their payments were directly proportional to their performance. If the supervisor observed their facial expressions, two yuan for each expression would be subtracted from their payment. On the contrary, if the supervisor did not observe any facial expressions, ten yuan would be added to their payment as an additional reward.

The seven video clips were grouped into three blocks. Each block comprised three positive, one neutral, and three negative videos played sequentially. To avoid order effects, the order of playing the positive and negative video clips was reversed for different participants. Before starting the experiment, 60 s of resting state was collected as the baseline. After each video, participants were allowed to rest for 60 s and their emotional activation were measured with a 7-point Likert scale. The average score for negative materials was 5.91 and that for positive materials was 6.11.

### Data collection, processing, and analysis

#### Facial expression identification and processing

**FIGURE 2:**
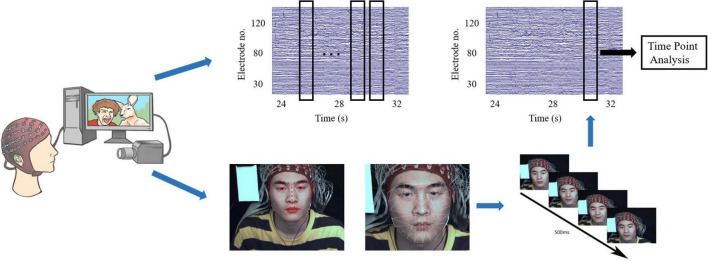
Diagram of facial expression identification and determination of the time points of micro-expressions.

Complete micro-expressions are difficult to elicit in the laboratory, and only partial facial expressions, such as movements from the lower or upper face, are typically observed (Porter et al., 2012; Yan et al., 2013). However, partial micro-expressions tend to be subtle manifestations of an underlying emotion, and observations of rapid facial expressions in various social situations indicate that micro-expressions are more often partial than are full-face expressions. In this study, partial or full facial expressions with durations of 500 ms were considered micro-expressions, whereas those with durations > 500 ms were classified as macro-expressions. We first used action units (AUs) from a facial action coding system (Yan et al., 2014) to detect micro-expressions among participants at the same stimulus time points based on a discriminative response map fitting method (Asthana et al., 2013), tracking 66 facial landmarks of facial expressions (Liu et al., 2016). Two coders were used to analyze the micro-expressions (Yan et al., 2014). The procedure includes the following three steps (see Figure 2).

Step 1: Count the time points of all micro-expressions across all participants. This step was used to detect all the time points of the participants’ micro-expressions when they watched the videos. We determined the approximate time points for the onset, apex, and offset frames by playing the recording at a 1/3 speed. We selected all time points based on these results.
Step 2: Frame-by-frame coding. This step was used to determine the onset, apex, and offset frames of the micro-expressions based on the time points previously selected. As micro-expressions under positive emotion for instance, the first frame that showed activation of AU6, AU12 (or both) was considered the onset frame. For this facial expression, the apex frame displayed the entire expression with the maximum intensity. The offset frame is the last frame before the face reverts to its original expression (Hess and Kleck, 1990; Hoffmann, 2010; Yan et al., 2013). The coders repeatedly examined minute changes between adjacent frames surrounding the micro-expression onset, apex, and offset to identify these frames accurately. The duration of micro-expression was calculated.
Step 3: Determining time points: Based on the apex frame, we selected time points when > 20 participants showed micro-expressions. Global time points were determined by averaging the timestamps of the apex frames. Based on these reference points, 2-s blocks of the corresponding EEG signals were extracted for each participant to perform the analysis. Participants with micro-expressions occurring at these time points were included in the micro-expression group.

#### Electroencephalography signal processing and analysis

##### Acquisition and pre-processing

Electroencephalography was recorded continuously from 128 electrodes using an ActiveTwo acquisition 125 system (BioSemi, The Netherlands) at a sampling rate of 2,048 Hz. EEG data were processed offline using EEGLAB (Delorme and Makeig, 2004) and BESA research software (Hoechstetter et al., 2004). Drift and noise reduction were performed by applying a 0.5-50 band-pass filter. To maximize the signal-to-noise ratio, the EEG signals should be referenced to the Reference Electrode Standardization Technique (REST) reference using the REST software (Yao, 2001; Dong et al., 2017). We used BESA research software to correct EEG signals contaminated by eye blinks and movements.

The time synchronization signal and timing of the apex frame of the facial expression were used to estimate the position of the EEG signal. We found that the EEG responses of micro-expressions were relatively short, generally within 1 s, and macro-expressions generally longer. Thus, with the apex of all facial expressions as the midpoint, we trimmed segments with a length of 2 s from the pre-processed EEG signals. The global field power (GFP) (Khanna et al., 2015) was subsequently calculated for the 2 s segments. We selected 1 s of data with the maximum peak (calculated using GFP) as the midpoint for the sample. GFP represents the strength of the electric field over the brain at each instant, and is often used to characterize rapid changes in brain activity. We selected the data with the maximum peak as the midpoint because the peak of the GFP curve instantaneously represented the strongest field strength.

##### Scalp-level analysis

To identify the average power spectral density (PSD) value in different frequency bands for each condition (occurrence of micro- and macro-expressions in positive/negative emotions), we extracted EEG signals using the open-source MATLAB toolbox FieldTrip (Oostenveld et al., 2011). The PSD at each electrode was calculated in 1 Hz steps between 0.5 Hz and 50 Hz with a seven-cycle-length sliding window. This resulted in a decreasing time window length as the frequency increased (e.g., 700 ms for 10 Hz and 350 ms for 20 Hz). The average PSD values from the alpha (8 Hz < f < 12 Hz), beta (12 Hz < f < 30 Hz), and gamma (30 Hz < f < 50 Hz) bands were extracted for all 128 electrodes.

The possible activity among electrodes at different frequency points associated with the micro- and macro-expressions was evaluated by calculating the differences in each condition at each frequency point. The resulting differences were first compared using a *post hoc* independent-sample t-test to compare the changes elicited by the occurrence of micro-expressions with macro-expressions. We used multiple-comparison corrections based on randomization statistical non-parametric mapping (5,000 times) to assess the statistical significance of all t-values (*p* < 0.05) (Maris and Oostenveld, 2007).

##### Source-level analysis

We used standardized low-resolution brain electromagnetic tomography (SLORETA) analysis to localize the sources of the difference between the two types of facial expressions induced by changes in brain oscillations (Fuchs et al., 2002; Pascual-Marqui, 2002; Jurcak et al., 2007). This calculates the cortical three-dimensional distribution of current source density (CSD) for micro-expressions and macro-expressions. For this purpose, scalp activity recorded at each electrode was first converted into the current CSD field on a three-dimensional source space (6,239 cerebrospinal gray matter voxels with a resolution of 5 mm) based on the transformation matrix. Subsequently, voxel-by-voxel one-sample t-tests on log-transformed data between the two conditions were used to determine whether these two larger late positivities would be mediated by distinct functional neural structures. Multiple comparison corrections based on randomization statistical non-parametric mapping (5,000 times) were used to assess the statistical significance of all t-values (*p* < 0.05).

## Results

**FIGURE 3:**
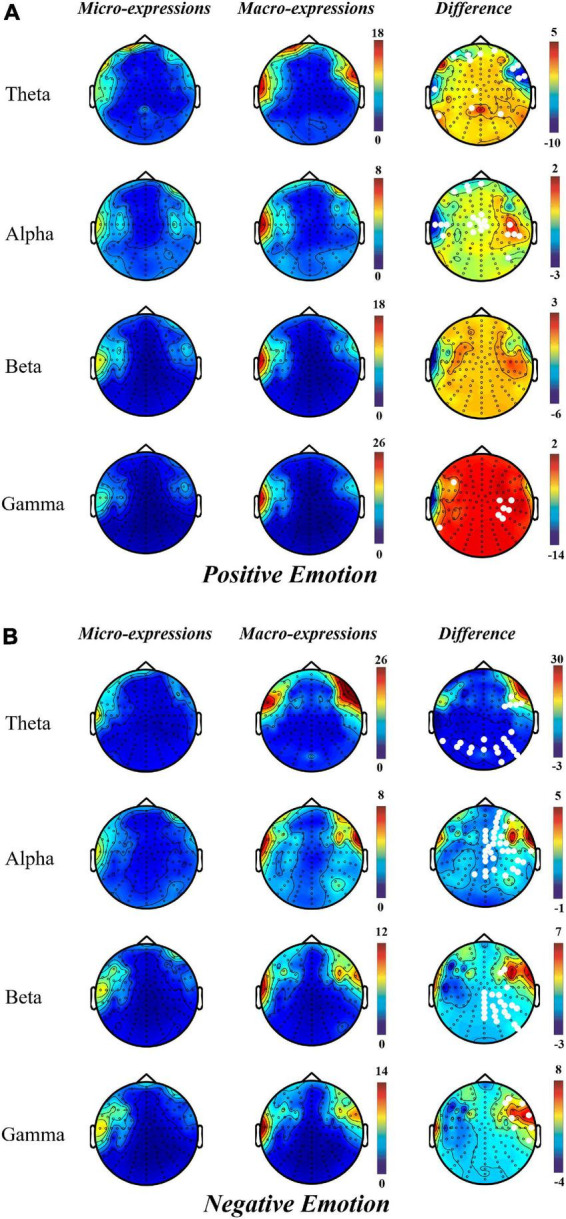
Topographical maps for EEG power in micro-expression and macro-expression from theta, alpha, beta and gamma bands in **(A)** Positive emotion and (Micro-expression minus Macro-expression) **(B)** Negative emotion (Macro-expression minus Micro-expression). The white dots on the difference graph are the electrode points with significant difference between the power of micro-expression and macro-expression.

The results of topographical maps showed that, during positive emotion, compared with macro-expression, the power of micro-expression was significantly higher in the right central and parietal regions in the alpha and gamma bands. However, in the theta and alpha bands of the prefrontal and left temporal regions, micro-expressions had lower power than macro-expressions (Figure 3A).

Contrary to the pattern of positive emotion, the power of macro-expressions in negative emotion was greater than that of micro-expression; significant differences were located in the channel from the parietal and occipital regions in theta bands; right prefrontal, right frontal, right central, right parietal, and right temporal regions in the alpha band; right frontal, right central, right parietal, and right parietal regions in the beta band; and right frontal, right central, and right temporal regions in the gamma band (Figure 3B).

Micro- and macro-expressions have a similar activation pattern in positive and negative emotions. This is more prominent in the left prefrontal and right frontal regions in the theta and alpha bands and left temporal region in the theta, alpha, and gamma bands.

**FIGURE 4:**
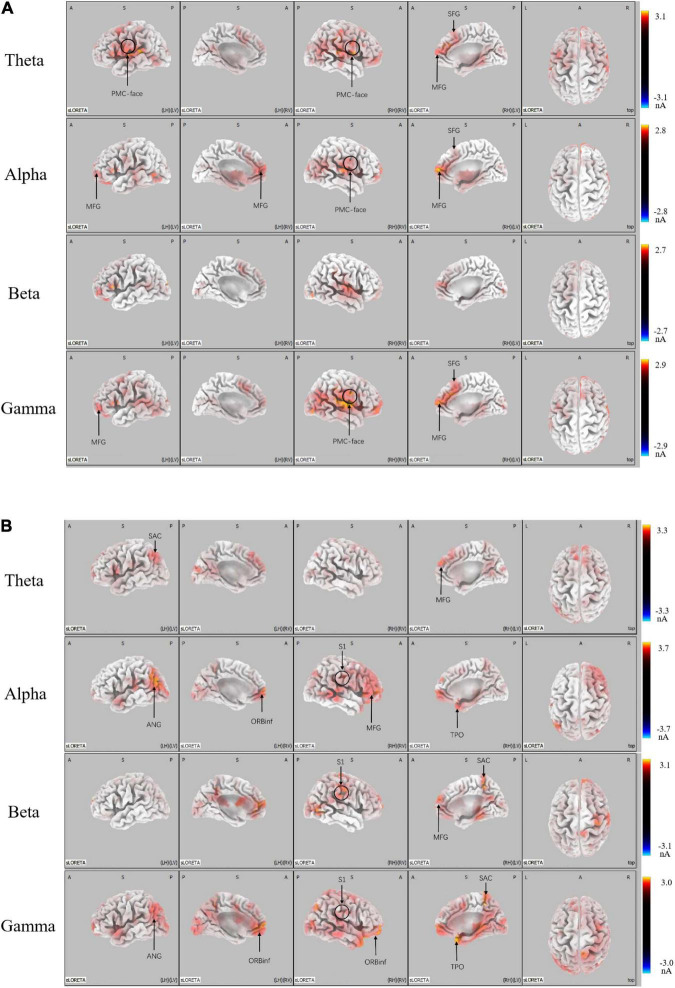
LORETA probabilistic map showing cortical activation and a significant difference between micro-expression minus macro-expression in **(A)** Positive emotion and **(B)** Negative emotion (Micro-expression minus Macro-expression) **(B)** Negative emotion (Macro-expression minus Micro-expression). Red colors represent a greater activation, blue colors represent a less activation.

We conducted the SLORETA analysis to further determine whether the brain areas were mediated by distinct functional neural generators. There was greater activation in micro-expression (p < 0.05) for a positive emotion (see Figure 4A). We found significant differences in: the theta band located in the promoter cortex (PMC), right superior frontal gyrus (SFG), and right middle frontal gyrus (MFG); alpha band, which is also located in PMC, right SFG, and MFG; and gamma band, which is located in the PMC, MFG, and SFG. There was greater activation in macro-expression (p < 0.05) for a negative emotion (Figure 4B). We found significant differences in: the theta band located in the left somatosensory association cortex (SAC) in the occipital region; alpha band, which is located in the MFG in the prefrontal region, somatosensory cortex (S1) in the right central region, left angular gyrus (ANG) in the occipital region, and temporal pole (TPO) in the temporal region; beta band, which is located in the SAC in the occipital region, and S1 in the right central region; and gamma band located in the SAC and ANG in the occipital region, S1 in the right central region, and TPO in the temporal region (see Figure 4B).

**FIGURE 5:**
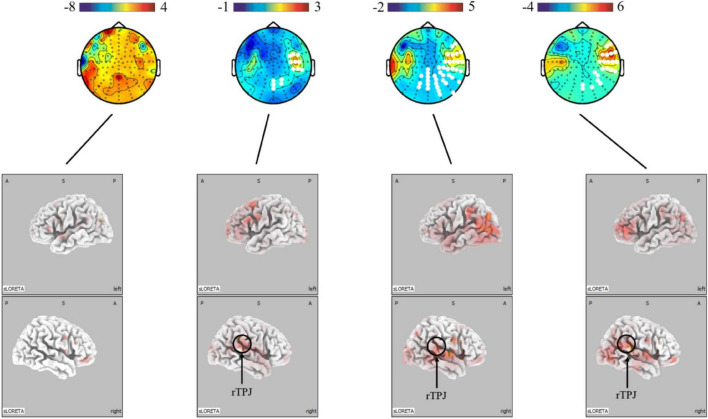
Topographical maps for EEG power and LORETA probabilistic map showing cortical activation and a significant difference between micro-expression under positive emotion and negative emotion (positive emotion minus negative emotion). Red colors represent a greater activation, blue colors represent a less activation. The white dots are the electrode points with significant difference.

The present study further analyzed the differences in micro-expressions under positive and negative emotions. The power under positive emotion in the alpha, beta, and gamma bands on the scalp level was greater in the right parietal, temporal, and occipital regions than under negative emotion. This difference was not significant in the theta band. The source-level results showed stronger activation under positive emotion in the right temporoparietal junction (rTPJ), which is associated with attentional control (see Figure 5).

## Discussion

In order to help overcome for the limitations of micro-expression expert assessment and image recognition techniques, and lay the foundation for micro-expression recognition based on EEG signals. This study is the first to investigate the brain mechanisms of micro-expressions and their differences from macro-expressions from a neuroscience perspective. As a result, we found (1) a general activation of the left temporal and frontal regions in micro-expressions and macro-expressions under both positive and negative emotions; (2) that under positive emotion, micro-expressions shows stronger power in the central and parietal regions on the scalp in the alpha and gamma bands than macro-expressions, which are located in the premotor, supplementary motor cortices, and MFG on the source level. and (3) that under negative emotion, the micro-expressions showed lower power in the right prefrontal, right frontal, right central, right parietal, and right temporal regions than macro-expressions across frequencies in negative emotion. At the source level, differences were located in the somatosensory cortex and angular gyrus regions. These three main points are elucidated in the following discussion.

We found a similar activation on the scalp for micro- and macro-expressions under both positive and negative emotions, with general activation in the left temporal and prefrontal regions, which concurs with previous emotional suppression studies (Ochsner et al., 2012; Buhle et al., 2014). In the present study, participants in the SEES were requested to perform neutral facial expressions that conflicted with their real emotional state. Activation of the prefrontal region, especially higher power in the alpha band (Pfurtscheller et al., 1996; Klimesch, 1999; Coan and Allen, 2004), was reported to be associated with voluntary control of social-emotional behavior and involved in coordinating rapid action selection processes, emotional conflict detection, and inhibition of emotional responses (Volman et al., 2011; Roy et al., 2012; Rive et al., 2013). For instance, in an emotional conflict task (face-word task), successful emotional conflict resolution was associated with regions of the ventral medial prefrontal cortex, cortex supplementary motor area, and superior temporal gyrus (Deng et al., 2014). Meta-analyses of fMRI showed that response inhibition activated the frontostriatal system, including the ventral lateral prefrontal cortex and supplementary motor areas (Hung et al., 2018). In addition, temporal regions are core regions for emotion detection, and the left temporal lobe is considered to be involved in the action component of emotion (Bamford et al., 2009; Jastorff et al., 2016), in avoiding negative items. In addition, according to recent research, activation of the superior temporal cortex suggests that the extrastriate and temporal cortices (such as the superior temporal sulcus) are relevant to facial processing (Gerbella et al., 2019). There are reports in patients with schizophrenia that temporal lobe structural abnormalities are related to deficits in facial emotion recognition (Goghari et al., 2011). Considering both these findings, the general activation in the frontal and left temporal regions appears to reflect the participants’ inhibition state of facial control for emotional stimulation and/or responses when suppressing emotional facial expressions.

For positive emotions, the premotor area showed a significant difference, while micro-expressions showed higher activation in this area. Higher activation of the premotor cortex correlates with facial expression control, as the premotor cortex, supplementary motor area, and primary somatosensory cortex are key regions controlling all subdivisions of the facial nucleus and implicate sensorimotor simulation during action observation (Grosbras and Paus, 2006; Majdandzic et al., 2009). For instance, direct electrical stimulation of the rostral portion of the supplementary motor area elicits complex facial movement patterns (Fried et al., 1991). Furthermore, the premotor cortex is more directly implicated in emotion-related motor behavior, which has been often reported in studies on the passive observation of actions (Balconi and Bortolotti, 2013; Mattavelli et al., 2016). The prefrontal cortex and premotor cortex are the core regions that control responses to emotional cues (Coombes et al., 2012; Braadbaart et al., 2014; Perry et al., 2017). Activation of these areas has been interpreted as evidence that contributes to explicit emotion processing by linking emotion perception with representations of somatic states engendered by emotions (Balconi and Bortolotti, 2013). Furthermore, to support this viewpoint research has indicated that comprehending another person’s facial expressions is related to increased activity in similar sensorimotor cortices (Banissy et al., 2010; Karakale et al., 2019). In the present study, the higher activity of micro-expressions in the premotor cortex may reflect stronger engagement of face control in positive emotions.

In contrast, under negative emotions, macro-expressions were more strongly activated in the somatosensory cortex and angular gyrus regions than in micro-expressions. This may indicate a stronger emotional arousal of macro-expressions compared to micro-expressions of fear. A previous study on fear processing found preferential involvement of the somatosensory cortex in fear processing (Williams et al., 2004; Bertini and Ladavas, 2021). For instance, the prominent role of somatosensory cortices in previous studies on embodied emotional simulation showed consistent activation of somatosensory areas when observing and producing emotional facial expressions (Carr et al., 2003; Winston et al., 2003). Moreover, information regarding the presence of a potential threat relies more heavily on somatosensory representations than other emotions (Pourtois et al., 2004). Activation on the right side of the somatosensory cortex further demonstrates the function of recognizing fearful emotional expressions (Pourtois et al., 2004). For instance, lesion studies showed that damage to the right somatosensory cortex significantly impairs recognition of emotional facial expressions (Adolphs et al., 2000). Inhibiting the activity of the right somatosensory cortex with repetitive transcranial stimulation interferes with the embodied simulation mechanism, thereby disrupting the ability to recognize emotional facial expressions (Pitcher et al., 2008). More importantly, conscious visual perception of fear-related stimuli involves cortical visual pathways to the amygdala, including the primary and extrastriate visual areas (Morris et al., 1999; Cecere et al., 2014; Bertini et al., 2018). This concurs with our finding that the activation of ANG shows that fear-related salient visual stimuli elicit activity in defense circuits that can heighten visual perceptual processing (Keil et al., 2010). Therefore, the specific higher activation occurring in the macro-expressions seems to reflect a stronger facial representation elicited by fear. This has been attributed to a preliminary activation of the somatosensory cortices in response to threat due to a possible interaction between the networks subserving visual perception and emotional arousal mechanisms (Gall and Latoschik, 2020). Accordingly, the stronger activation of macro-expressions in the somatosensory cortex and angular gyrus regions and their right-side lateralization suggest a stronger sensation of fearful emotions in macro-expressions.

These findings, collectively, indicate that micro-expressions are different from macro-expressions. However, the differences are not the same for positive and negative emotions. A possible explanation for the difference between the positive and negative emotional conditions is that the face is controlled by two main pathways: cortical and subcortical (Haxby et al., 2000; Adolphs, 2002). The cortical pathway is top-down voluntary control, whereas the subcortical pathway is a bottom-up physiological response (Vuilleumier and Pourtois, 2007; Ishai, 2008). For instance, the amygdala can influence the activity of somatosensory cortices in the presence of a potential threat, resulting in increased activity in the sensorimotor system and direct projection to the facial nucleus (Bertini and Ladavas, 2021). Thus, when we compared the differences between macro-expressions and micro-expressions under negative emotions, owing to the subcortical way, we found that macro-expressions had stronger activation on the somatosensory and visual cortices. In contrast, in facial expressions of happiness, smiles required little preparation and were usually easier to control in accordance with the requirements. Thus, voluntary control of micro-expressions of positive emotions in the cortical pathway can be observed in the premotor cortex. In other words, micro- and macro-expressions exhibit differences in neural activity under the same conditions; however, these differences vary under positive and negative emotions. Moreover, micro-expressions under positive emotion were more strongly activated in the right temporoparietal junction (rTPJ) than in negative emotion. A previous study found that stronger activation of the rTPJ was associated with greater attentional control. For instance, it influences the detection of deviant stimuli in oddball paradigms (Arrington et al., 2000; Jakobs et al., 2009). Taken together, this is because the pathways of facial control are different; the production of micro-expressions under positive emotions is dependent on the control of the face, while micro-expressions under negative emotions are more dependent on the intensity of the emotion.

This study had several limitations. We used the dipole-source method to study neural activities associated with the generation and evaluation of significant differences between micro-expressions and macro-expressions. However, we were unable to detect the neural activity of key emotion processing structures (such as the amygdala) buried deep in the lower cortex, because of the inherent deficiency in EEG detection depth. Therefore, we were unable to grasp the complete picture of the brain mechanisms involved in micro-expression. Higher spatial resolution tools such as fMRI are needed for future research.

## Conclusion

This study is the first to investigate the neural mechanisms underlying the differences between micro- and macro-expressions by using EEG signal. It helps to fill the gap in the field of identifying micro-expressions in neural activation. We designed a paradigm SEES, which we believe is a worthy contribution to the elicitation of micro-expressions. Our findings highlight that both micro- and macro-expressions activate the left temporal and prefrontal lobes under different emotions. This reflects the participants’ state of inhibition of facial control in response to emotional stimuli and/or responses to emotional facial expressions. Micro-expressions were strongly activated in the premotor cortex, supplementary motor cortex, and MFG regions under positive emotions. They were more weakly activated in the somatosensory cortex and angular gyrus regions under negative emotions. This indicates that micro-expressions under positive emotions are dependent on the control of the face, whereas under negative emotions, they are dependent on the intensity of the emotion. Our findings highlight that this difference occurs because the pathways of facial control are different, which contributes to the groundwork for future research on the mechanism and pattern recognition of micro-expressions.

## Data availability statement

The original contributions presented in this study are included in the article/supplementary material, further inquiries can be directed to the corresponding author.

## Ethics statement

The studies involving human participants were reviewed and approved by the Ethical Review Committee of Southwest University. The patients/participants provided their written informed consent to participate in this study. Written informed consent was obtained from the individual(s) for the publication of any potentially identifiable images or data included in this article.

## Author contributions

All authors listed have made a substantial, direct, and intellectual contribution to the work, and approved it for publication.
